# Novel pectin-based nanocomposite film for active food packaging applications

**DOI:** 10.1038/s41598-022-25192-4

**Published:** 2022-11-30

**Authors:** Muhammed R. Sharaby, Emad A. Soliman, Adel B. Abdel-Rahman, Ahmed Osman, Rowaida Khalil

**Affiliations:** 1 Basic and Applied Sciences (BAS) Institute, Egypt-Japan University of Science and Technology (E-JUST), New Borg El-Arab City, Alexandria, 21934 Egypt; 2 Botany and Microbiology Department, Faculty of Science, Alexandria University, Alexandria, 21511 Egypt; 3 Polymeric Materials Research Department, Advanced Technology and New Materials Research Institute (ATNMRI), City of Scientific Research and Technological Applications (SRTA-City), New Borg El-Arab City, Alexandria, 21934 Egypt; 4 Department of Electronics and Communications Engineering, Egypt-Japan University of Science and Technology, New Borg El-Arab City, Alexandria, 21934 Egypt; 5 Department of Biochemistry, Faculty of Science, Ain Shams University, Cairo, Egypt

**Keywords:** Biotechnology, Microbiology

## Abstract

Novel pectin-based films reinforced with crystalline nanocellulose (CNC) and activated with zinc oxide nanoparticles (ZnO NPs) were prepared by solvent-casting method. Film ingredients enhanced UV-blocking, thermal, and antibacterial properties of active films against well-known foodborne pathogens. Optimal active films exhibited higher mechanical, water vapor barrier properties compared to pristine pectin films. SEM confirmed the even distribution of CNC and ZnO NPs in pectin matrix and their interactions were proven using FTIR. Wrapping hard cheese samples artificially contaminated with *Staphylococcus aureus* and *Salmonella enterica* with the ternary nanocomposite film at 7 °C for 5 days significantly reduced the total population counts by at least 1.02 log CFU/g. Zn^2+^ migrating to wrapped cheese samples was below the specific limit (5 mg/kg), confirming their safety for food contact. Overall, ZnO/CNC/pectin nanocomposite films represent promising candidates for active food packaging as safe, eco-friendly alternatives for synthetic packaging materials.

## Introduction

Non-biodegradable synthetic plastics are commonly used for food packaging owing to their low cost, processability, good aesthetic quality, and excellent physicochemical properties. However, they have negative ecological impacts besides their health-related issues^1,2^. Increased consumers' awareness and concerns towards the environment have recently reflected on a higher demand to utilize eco-friendly biodegradable biopolymers such as gluten^3^, gelatin^4^, chitosan^5^, starch^6^ and alginate^7^ as alternatives to traditional plastic packaging materials.

Pectin; a heterogeneous polysaccharide found in plant cell walls, composed of α-(1,4)-linked D-galacturonic acid, is one of the extensively used polysaccharides for packaging films^8^. Pectin properties are influenced by the degree of methyl esterification, which varies according to plant origin and processing conditions^9^. Pectin’s biodegradability, biocompatibility, gelation properties, and non-toxicity highly favor its suitability as a food packaging material^10^; however, its moderate barrier, thermal and mechanical properties besides its high hydrophilic nature, may limit its applicability^11^. Recently, biopolymer-based active packaging systems have attracted the interest of both academia and industrial sectors for the purpose of maintaining food safety and quality. Functional properties of pristine pectin films may be enhanced by blending with other polymers^12–15^, or the addition of nanosized fillers^14,16,17^ that contribute to enhancing inherent film properties and improving physicochemical, barrier, thermal, and mechanical properties.

Lignocellulosic and cellulosic waste materials can be valorized as eco-friendly, sustainable, and low-cost precursors to produce crystalline nanocellulose (CNC) either by mechanical methods, enzymatic or acid hydrolysis^18^. CNC have been used to improve the properties of polymer matrices due to their intrinsic advantages of high surface area, low density, high mechanical strength, and availability^19^. For instance, improvement in the quality and shelf-life of ground meat and acerola cherries packaged with CNC-based composites have been reported by Azeredo et al. (2012)^20^ and Dehnad et al. (2014)^21^. Zinc oxide nanoparticles (ZnO NPs) are another promising filler materials with unique features such as strong antimicrobial activity (AMA), UV-light blocking properties, and high catalytic and photochemical activity^22^, and have low toxicity to biological systems. Moreover, ZnO NPs have been approved as transparent UV light absorbers in food packaging applications by the European Food Safety Authority^23^ and are generally recognized as safe (GRAS) material by the Food and Drug Administration (FDA) protocol (21CFR182.8991)^24^. To the best of our knowledge, there is no information evaluating the impact of the synergistic combination of ZnO NPs and CNC on reinforcing the properties of pectin-based films.

In the present study, active ZnO NPs/CNC/pectin-based films were prepared using the solvent casting method. Physicochemical, morphological, thermal, barrier and mechanical properties of pectin-based nanocomposite films were investigated. The AMA of nanocomposite films was studied in vitro and in artificially contaminated cheese samples stored at 7 °C for 5 days. Cytotoxicity and migration of Zn^2+^ from the ZnO NPs/CNC/pectin hybrid-based films to an extensively consumed cheese type were assessed upon wrapping to confirm the safety and potential applicability of the produced active films.

## Materials and methods

### Materials

Pectin (63–66% degree of esterification, Mw = 30,000–100,000 g/mol), polyethylene glycol 400 (PEG_400_), zinc nitrate, and magnesium nitrate hexahydrate were purchased from Loba Chemie, India. Anhydrous calcium chloride and sodium chloride were provided by Fisher Scientific, UK. Microbiological media, including Tryptic Soy Broth (TSB), Mueller–Hinton Agar (MH), Xylose Lysine Deoxycholate agar (XLD), and Mannitol Salt Agar (MSA), were provided by HiMedia (India). All solvents were of analytical grade and obtained from recognized chemical suppliers.

### Synthesis and characterization of ZnO NPs

ZnO NPs were prepared by co-precipitation method using zinc nitrate as a precursor and sodium hydroxide as a precipitating agent^25^. 100 mL of 0.5 M zinc nitrate solution was dissolved in distilled water (DW) at 70 °C for 1 h. Zinc nitrate solution was added dropwise to 1 M NaOH solution that was kept stirring (SMHS-3, Daihan Scientific Co., Korea) at 500 rpm till a white precipitate was formed. The mixture was allowed to settle at room temperature (RT) overnight, washed three times with DW and ethanol, and dried in a hot air oven at 70 °C for 1 h. The resulting powder was ground using a mortar into fine particles and calcinated in a muffle furnace (Hobersal HD-230, Spain) at 400 °C for 2 h.

### Preparation of CNC

CNC was prepared from cotton linter by acid hydrolysis according to our previous work^26^. The CNC's average dimensions were determined using transmission electron microscopy (TEM). Images were analyzed using Gatan Micrsoscopy suite software (Gatan Inc., version 2.02.800.0, Japan) and the average length and width of at least 30 crystals were found to be 205.0 ± 29.0 and 15.80 ± 3.50 nm, respectively.

### Preparation of pristine pectin and pectin-based nanocomposites film

**Figure 1:**
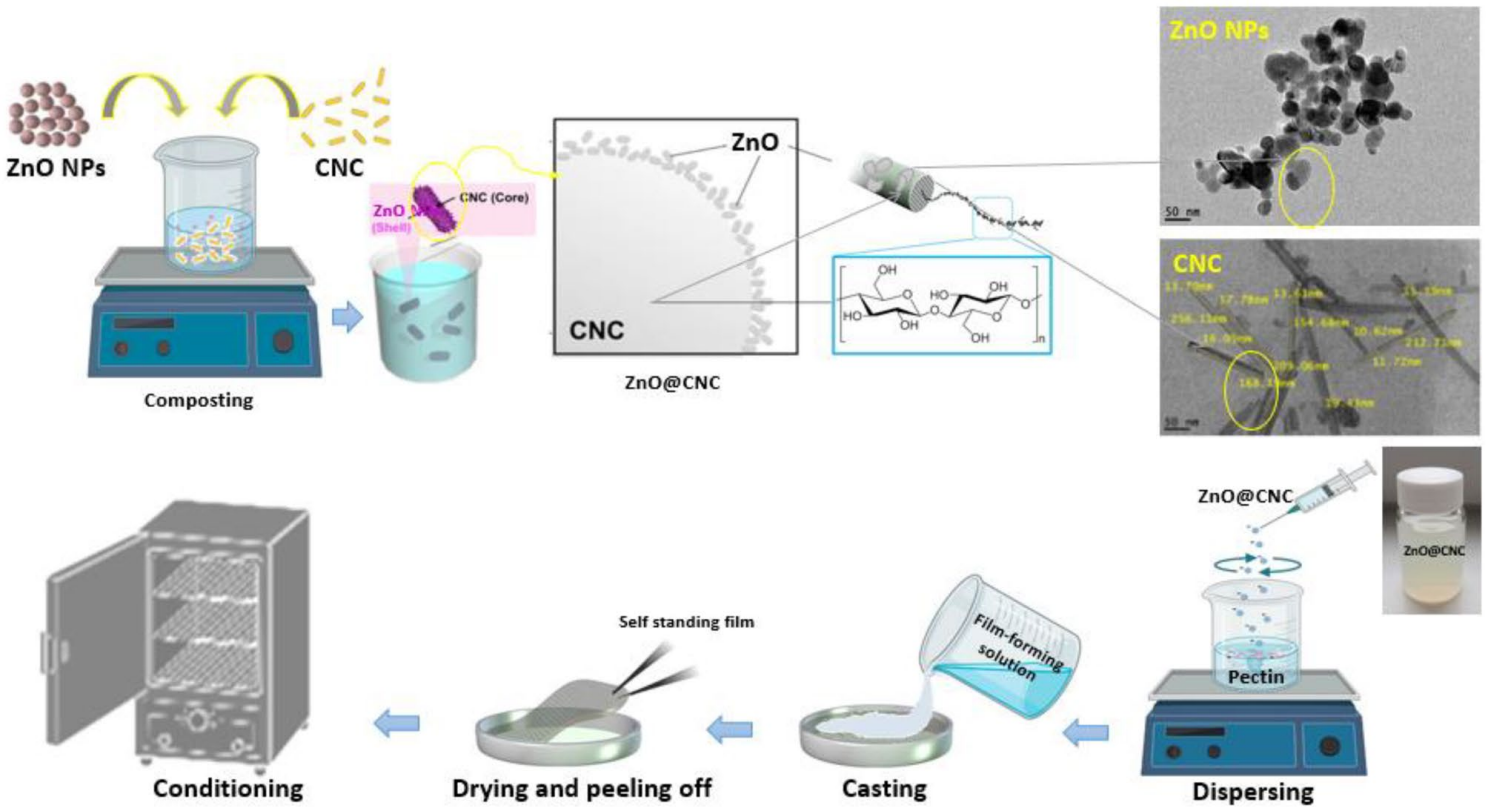
Schematic diagram for the preparation of ZnO/CNC composite and pectin-based nanocomposite films.

Pristine pectin (PC), CNC/pectin or ZnO NPs/CNC/pectin-based nanocomposite films were prepared according to Chaichi et al.^27^ by solvent-casting method with some modifications (Fig. 1). Pectin powder (3% w/v) was dispersed in 100 mL DW and heated at 70 °C on a hot plate until dissolution. PEG_400_ was added as a plasticizer at a concentration of 30% (w/w of pectin, pH was adjusted at 4.0, and the solution was further stirred for 1 h to give the PC film-forming solution (FFS). CNC suspension was prepared by suspending different concentrations of CNC (1, 3, 5 and 7% w/w of pectin) in 20 mL DW, sonication (WUC-D06H, Daihan Scientific Co., Korea) at 20 kHz for 30 min, followed by slow addition to the previous PC-FFS (80 mL) to give the CNC/PC-FFS. The ZnO NPs/CNC/PC ternary system was prepared by adding ZnO NPs solutions (0.5, 1, 2, or 3% w/w of pectin) to the previously prepared CNC suspension (5% w/w; optimum concentration) with constant stirring. The resulting ZnO/CNC suspension was slowly added to the previously prepared pectin solution and sonicated for 30 min to obtain the ZnO/CNC/PC-FFS. The prepared FFSs were degassed to remove air bubbles prior to casting onto polystyrene Petri dishes (14 cm diameter) and drying at RT for 24 h. All dried films (Table 1) were peeled off and conditioned at 53.0 ± 1% relative humidity (using saturated magnesium nitrate hexahydrate solution) at RT in a desiccator for 48 h till further characterization.

**Table 1:** Formulations of the prepared films. PEG400, CNC and ZnO NPS are added as % (w/w) of pectin per 100 mL solution.

Film ID	Formulations			
	Pectin (g)	PEG_400_	CNC	ZnO NPs
PC	3	30	–	–
CNC/PC I	3	30	1	–
CNC/PC II	3	30	3	–
CNC/PC III	3	30	5	–
CNC/PC IIII	3	30	7	–
ZnO/CNC/PC I	3	30	5	0.5
ZnO/CNC/PC II	3	30	5	1
ZnO/CNC/PC III	3	30	5	2
ZnO/CNC/PC IIII	3	30	5	3

### Characterization experiments

#### Antimicrobial and cytotoxic activities of FFSs

##### AMA

The AMA of the FFSs was evaluated using the well diffusion method^28^ against six different pathogenic bacterial strains (*E. coli* O157:H7, *Klebsiella pneumoniae, Proteus mirabilis*, *Pseudomonas aeruginosa, Salmonella enterica*, and *Staphylococcus aureus*). 100 µl aliquots of each bacterial suspension (1.5 × 10^7^ CFU/ml) was spread plated onto Muller-Hinton plates. Then, wells (6 mm in diameter) were formed in each plate into which 200 µl of each FFS was added. Plates were incubated at 37 °C for 24 h. The diameters of inhibition zones were measured and data was represented as means from three independent experiments.

##### Cytotoxicity

The cytotoxicity of the FFS of ZnO/CNC/PC III film was evaluated using the MMT (3-(4, 5 Dimethylthiazol-2-yl)-2, 5-diphenyltetrazolium bromide) colorimetric assay^29^. Human colorectal adenocarcinoma cells (Caco-2, HTB-37) and normal lung fibroblasts (WI-38, CCL-75) obtained from the American Type Culture Collection (ATCC, Rockville, MD); were seeded independently in flat-bottom 96 well plate (Corning, USA) at a density of 1 × 10^4^ cell/well (100 µL) in high glucose DMEM culture medium (Biosera, France) supplemented with 10% fetal bovine serum albumin (Sigma-Aldrich, USA) and 1% Penicillin streptomycin (Sigma Aldrich, USA). Plates were incubated at 37 °C for 18 h in a humidified 5% CO_2_ incubator (Eppendorf, Germany) to allow cells attachment. The FFS was serially two-fold diluted, added in triplicate in each well, and incubated for 48 h under the previously mentioned culture conditions. Supernatants from each well were withdrawn and replaced with fresh medium (100 µL/well) containing MTT reagent with a final concentration of 0.5 mg/mL, and plates were re-incubated at 37 °C for 4 h. Media over the cells were removed, and dimethyl sulfoxide (Merck, Germany) was added (100 µL/well), plates were shaken for 15 min, and the absorbance was measured at 570 nm using a microplate reader (AccuReader M965 + , Metertech, Taiwan).

### Characterization of ZnO NPs, pristine pectin and pectin-based nanocomposite films

#### Structural properties

##### Fourier transform infrared spectroscopy (FTIR)

The FTIR spectra of ZnO NPs, pristine pectin and pectin-based films were recorded in the frequency range of 4,000–400 cm^−1^ at a resolution of 4 cm^−1^ using FTIR spectrophotometer (Bruker vertex 70v, Germany).

##### X-ray diffractometry (XRD)

The crystallinity of ZnO NPs was investigated by an X-ray diffractometer (Shimadzu XRD-6100, Japan) using CuKα radiation generated at 40 kV and 40 mA at 2θ range between 5° and 90°. The average crystallite size of the prepared particles was calculated using Debye–Scherrer's formula (Eq. 1)

1 $$ D = \frac{0.89.\lambda }{{\beta .cos\theta }} $$

where $$ D$$ is the average size, $$\lambda $$ is the wavelength of CuKα (1.540 Å), $$\beta$$ is the full width at half maximum in radians, and $$\theta$$ is the Bragg diffraction angle.

### Morphological properties

#### TEM

The morphology and particle size of ZnO NPs were examined using TEM (JEOL JEM-2100F, Japan), with 1-A ° resolution. The operating voltage of the microscope was 200 kV.

##### Scanning electron microscopy (SEM)

The surface microstructure and cross-section of film specimens were examined using SEM (JEOL, JSM-6010LV) under vacuum conditions and an acceleration voltage of 20 kV. Film samples were sputter-coated (JEOL, JEC-3000FC) with gold to enhance the surface conductivity.

##### Optical properties

The absorption spectrum of ZnO NPs was recorded using a UV–Vis spectrophotometer (Hitachi U-3900, Japan) within the range of 200–800 nm. Film samples were cut into a rectangular piece and their UV-barrier property and opacity of the films were evaluated by measuring light transmittance at 280 nm (T_280_) and 600 (T_600_) and absorbance at 600 nm (A_600_), respectively. Film opacity was calculated^30,31^ by the following equation (Eq. 2)

2 $$ Opacity = \frac{A600}{x} $$

where *A*_*600*_ is the absorbance at 600 nm, and *x* is the film thickness (mm).

##### Mechanical properties

The thickness of the prepared films was determined using a digital micrometer (REXBETI, China). Ten random readings were taken per film, the mean thickness value was recorded and the test was repeated three times. Tensile strength (TS) and elongation at break (E%) were characterized using a universal electronic strength tester (Tensolab 5000, Mesdan, Italy) according to the standard method D882-12^32^. Sample films were cut into 2 cm × 8 cm strips. The initial grip spacing was 4 cm, and the crosshead speed was adjusted at 1 mm/s. TS was calculated by dividing the ultimate force by the cross-sectional area of each film. E% was calculated by the tester's software, and an average of five readings was recorded per sample^29^. The analysis of the stress vs strain curves was used to determine the Young's modulus (YM) and the integration of the area of each curve provided the stored energy until the breaking point (toughness)^33^.

##### Water barrier properties

Water vapor permeability (WVP) was determined gravimetrically at 25 °C according to ASTM E96-00^34^ with slight modifications. Film specimens were mounted on porcelain cups containing 8 g anhydrous calcium chloride, sealed with molten wax, cups were weighed and stored in a desiccator containing saturated magnesium nitrate solution (RH = 53.0 ± 1). The weight gain of each cup was monitored every 12 h for 5 days using a 4-digits balance (AXIS ACE220, Poland). Water vapor transmission rate (WVTR) and WVP were calculated from the following equations (Eqs. 3 & 4)

3 $$ WVTR = \frac{w}{t.A} $$

4 $$ WVP = \frac{WVTR. x}{{P_{o} . \left( {RH_{1} - RH_{2} } \right)}} $$

where *w/t* is the slope of weight gain line as a function of time (g/s) (calculated by linear regression with *R*^*2*^> 0.99), *A* is the area of the cup opening (m^2^), *x* is the average thickness of film (m), *P*_*o*_ is the partial pressure of water vapor at 25 °C (3169 Pa), and (*RH*_*1*_*–RH*_*2*_) is the relative humidity difference on the two sides of the film.

##### Moisture content (MC) and water absorption (WA)

MC and WA were determined by the gravimetric method^35^. Film specimens (2 cm × 2 cm) were weighed (*W*_*i*_), oven-dried at 105 °C for 24 h, weighed again (*W*_*f*_), and MC was determined by the following equation (Eq. 5)

5 $$ MC \% = \left( {W_{i} - W_{f} } \right)/W_{i} \times 100 $$

To determine the WA, the pre-dried film specimens were placed in a desiccator with saturated NaCl solution (RH = 75.0%). Films were daily weighed until a constant weight was reached (*W*_*f*_), and WA was calculated from the following equation (Eq. 6):

6 $$ WA \% = \frac{{\left( {W_{f} - W_{i} } \right)}}{{W_{i} }} \times 100 $$

##### Thermal stability

The thermal stability of the resulting films (⁓10–12 mg) were analyzed by thermogravimetric analyzer (Linseis STA PT1600/1000/LT, Germany) at ambient temperature up to 700 °C and a rate of 10 °C/min under a nitrogen purge at a flow rate of 60 and 40 mL/min for the balance and sample, respectively.

### Migration study

Wrapped cheese (Roumy; a traditional Egyptian hard cheese) and control samples (unwrapped cheese) stored at 7 ± 1 °C for 5 days were combusted at 550 °C in a furnace, and then the ash was dissolved in 65% (v/v) nitric acid^36^. A calibration curve was obtained using Zn^2+^ standard solutions at different concentrations diluted with HNO_3_ (1% v/v). The migrated Zn^2+^ from the active film to cheese samples were analyzed using an atomic absorption spectrometer (Agilent 280Z AA, Santa Clara, USA), and readings were presented as means ± SD (mg/Kg of cheese) of three determinations per sample.

### Wrapping experiment

The efficiency of the optimal active pectin-based film (ZnO/CNC/PC III) in inhibiting the growth of *S. aureus* and *S. enterica* populations in artificially contaminated cheese were assessed and compared to those detected in samples wrapped with pristine and LDPE films. Roumy cheese samples (3 cm × 3 cm) were surface sterilized under a UV lamp in a biosafety cabinet (BSC, Labconco A2, USA) for 15 min. 20 µL of each bacterial suspension (ca. 6.5 log CFU/mL) in TSB broth, were used to independently inoculate the upper surface of the sterilized cheese samples. Samples were left in the BSC for 20 min to allow bacterial attachment, wrapped with either PC, ZnO/CNC/PC III or LDPE films. Wrapped cheese samples were packed in polystyrene boxes, stored at 7 ± 1 °C for 5 days to mimic storage conditions at retail level, and samples were periodically removed for microbiological analysis. Each sample was homogenized using a 400C stomacher (Seward, UK) at 260 rpm for 2 min with sterile peptone water (0.1% w/v). Homogenates were serially ten-fold diluted, spread plated over selective media (XLD for *Salmonella* and MSA for *S. aureus*), plates were incubated at 37 °C for 24 h, and counts were presented as means ± standard deviation (SD) (log CFU/g) from three independent experiments^37^.

### Statistical analysis

Statistical analysis was performed with a completely randomized design with an analysis of variance (ANOVA) using OriginPro 2021 (Northampton, USA) software. All results were presented as means of at least two replicates from three independent experiments (unless otherwise stated) ± standard deviation (SD), where Tukey’s test was used to determine the significance of differences among the mean values at a *p* < 0.05 level.

### Results and discussion

#### Structural, optical, and morphological characteristics of ZnO NPs

**Figure 2:**
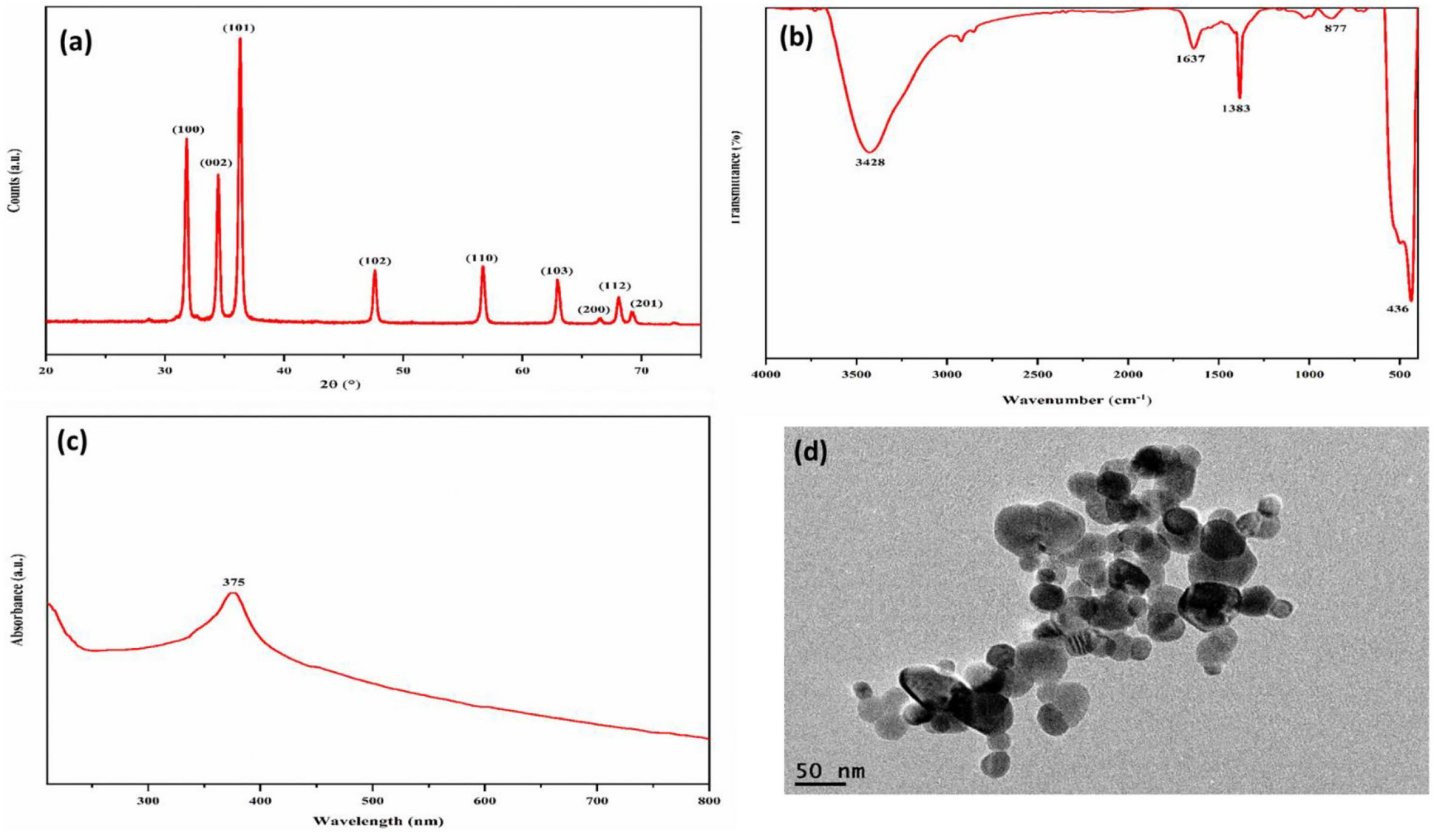
XRD pattern (**a**), FTIR spectrum (**b**), UV spectrum (**c**) and TEM micrograph (**d**) of prepared ZnO NPs.

The XRD pattern of synthesized ZnO NPs is shown in Fig. 2a Well-defined peaks at 2θ values of 31.81°, 34.47°, 36.30°, 47.61°, 56.72°, 62.99°, 66.51°, 68.11°, and 69.23° corresponding to lattice planes (100), (002), (101), (102), (110), (103), (200), (112), and (201), indicated the hexagonal wurtzite structures of ZnO as identified by the standard powder diffraction card (JCPDS Card no. 36–1451)^38^. The average crystallite size of ZnO NPs (24.73 nm) was calculated by OriginPro software using Debye–Scherrer's equation.

The FTIR spectrum of ZnO NPs in Fig. 2b revealed a characteristic absorption band at ⁓ 436 cm^−1^ assigned to the stretching vibration of the Zn–O bond in tetrahedral coordination^39^. A broad absorption peak appeared at 3428 cm^−1^ due to the O–H stretching vibration of water molecules. Absorption peaks at 1637 and 1383 cm^−1^ were ascribed to O–H stretching and bending vibration^40^, whereas the band at 877 cm^−1^ can be attributed to the asymmetric stretching vibration of C=O.

The optical absorption spectra of ZnO NPs were recorded using UV–vis spectroscopy in the 280–800 nm range. Figure 2c shows a strong absorbance peak at 374 nm corresponding to the characteristic band of pure ZnO^29^. Morphological features of the prepared NPs examined using TEM (Fig. 2d) revealed a quasi-spherical shape for most synthesized NPs with diameters ranging from ⁓17–40 nm. Variations in particle size may be linked to differences in nucleation time and NPs growth during the synthesis process. Slight agglomeration of particles was also noticed as a result of the large specific surface area and high surface energy of ZnO NPs^41^.

### Antimicrobial and cytotoxic activities of FFSs

#### AMA

The FFSs were assessed for their AMA against six foodborne pathogens using the well diffusion method (Table S1). PC and CNC/PC nanocomposite FFSs showed no activity against all tested pathogens. ZnO/CNC/PC nanocomposite FFSs exhibited higher potency against the Gram-positive; *Staphylococcus aureus* compared to Gram-negative pathogens, even at the least ZnO NPs concentration (0.5% w/w), because of the structural differences in cell wall composition of Gram-positive and negative bacteria in agreement with several reports^42–44^.

##### Cytotoxicity assay

Cytotoxicity assessment of ZnO NPs and CNC is crucial to ensure their health-related safety before their incorporation into food contact materials. IC_50_ value (dose of FFS required to kill 50% of cells) was evaluated using the MTT assay. IC_50_ values against cancerous Caco-2 and normal WI-38 were recorded at FFS concentrations of 4.53, and 24.61% (corresponding to 27.18 and 145.0 µg ZnO/mL, respectively). ZnO NPs have been reported to possess a higher selective cytotoxic activity against cancerous than their normal counterparts^45,46^. The cell mortality for both normal and cancerous cells was dose-dependent, as confirmed by the altered morphologies and abnormal shapes of the cells treated with higher ZnO NPs concentrations (Fig. S1). In accordance with several reports^47–49^, CNC tend to have no or very low toxicity to both normal and cancerous cell lines at similar concentrations used in the study (1.5 mg/mL). Furthermore, pectin is also known to possess no cytotoxic activities against normal cell lines^50–52^.

### Characterization of pristine and active pectin films

#### FTIR spectra

**Figure 3:**
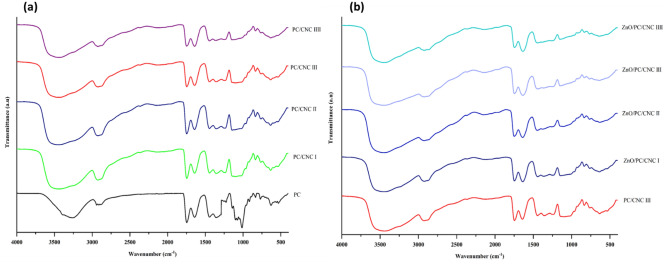
FTIR spectra of PC, CNC/PC (**a**) and ZnO/CNC/PC (**b**) composite films.

Characteristic absorption peaks of PC film (Fig. 3) were assigned to stretching vibration of O–H at 3260 cm^−1^, stretching of C–H at 2940 cm^−1^. The intense peaks at 1742 and 1643 cm^−1^ due to stretching vibration of methylated and non-methylated carboxylic groups, respectively, indicate the high methoxylation of pectin. The peak at 1141 cm^−1^ denoted the stretching vibration of C–O–C (glycosidic bond) belonging to the saccharide structure^10,53^. Increasing the amount of CNC in PC/CNC composite films resulted in an increased intensity and width of the O–H peak and a shift to a higher wavenumber (Fig. 3a), suggesting the possible formation of hydrogen bonding between CNC and pectin^54^. A less intense peak was observed at ⁓1740 cm^−1^, possibly due to the enhancement and reorientation of the hydrogen bonding in the pectin matrix^55^.

Figure 3b shows no significant changes upon the addition of ZnO NPs to the PC/CNC III composite; however, a slight shift in O–H peak at ⁓3450 cm^−1^ and a decrease in peak intensity at ⁓1747 cm^−1^ were recorded, which was attributed to the participation of the carboxylic group in hydrogen bonds^56,57^. It is speculated that the chemical structure of the polymer matrix was not modified by the addition of nanofillers, as confirmed by the absence of new absorption peaks. Hence, the reinforcing effect of the nanofillers was in the form of electrostatic interaction and hydrogen bonding between the filler and the pectin^54,58^.

#### Film morphology

**Figure 4:**
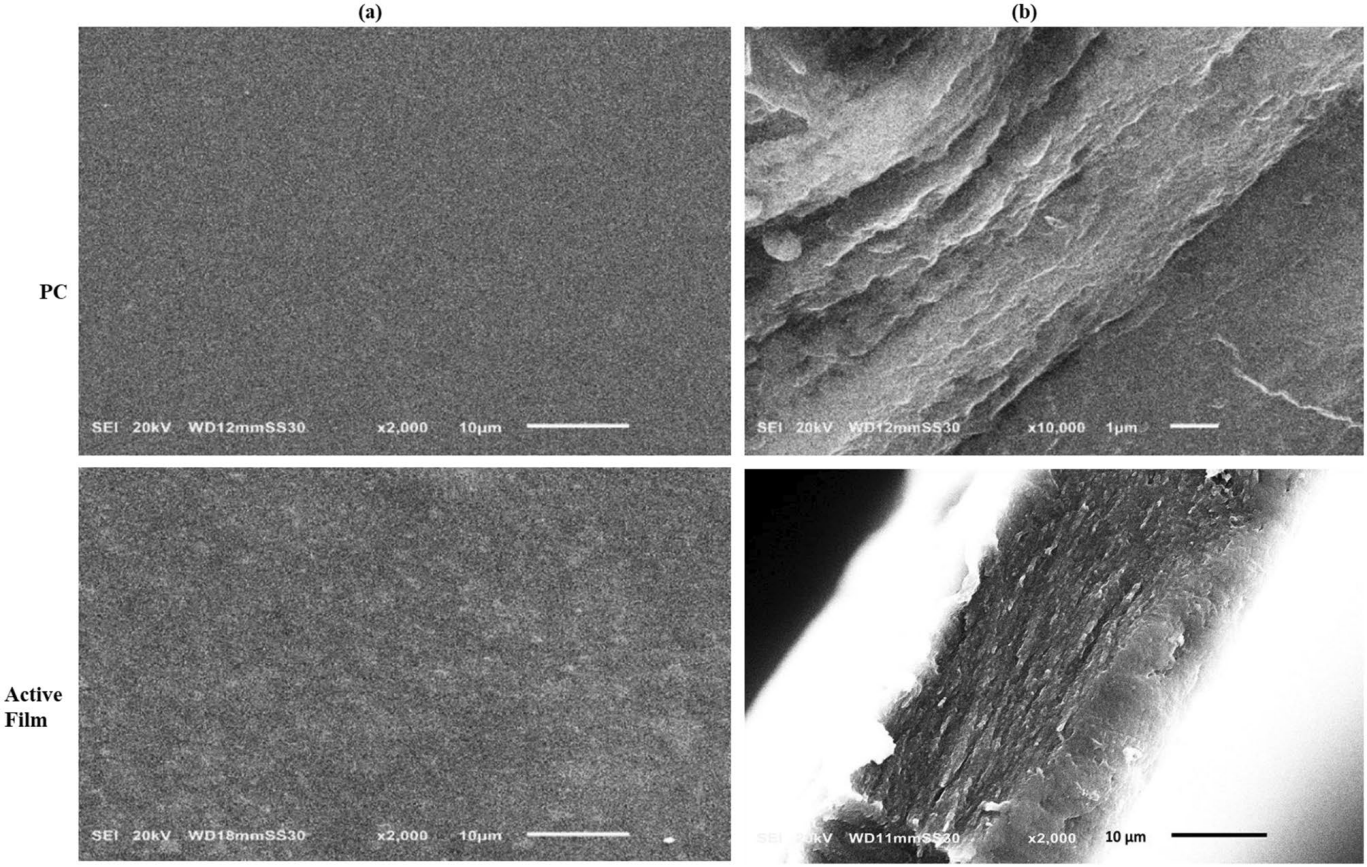
Surface morphology (**a**) and cross-sections (**b**) of SEM micrographs of PC and active film, respectively.

Surface and cross-section images of the PC and ZnO/CNC/ PC III films were examined using SEM (Fig. 4). PC film exhibited a smooth, homogenous surface with no pores or cracks (Fig. 4a), inferring the cohesiveness of the plasticized pectin matrix. In contrast, the micrograph of ZnO NPs/CNC/PC III nanocomposite film showed a rough and granular surface with uniform dispersion of nanostructured ZnO/CNC nanocomposite because of the high colloidal stability of ZnO/CNC nanocomposite in the FFSs^59^. The cross-section micrograph of PC film (Fig. 4b) showed a more loose and smooth surface than the nanocomposite-based film. Similar observations were found by Ahmadi et al.^60^ and Khan et al.^61^ for ZnO NPs/CNF/gelatin, and CNC/chitosan nanocomposite, respectively. In general, our findings confirmed the good dispersion and strong interfacial interactions between the nanocomposite filler and the polymer matrix.

#### Light transmission and opacity

The transparency of food packaging materials is recommended to allow the visibility and acceptance of products by consumers, whereas the UV-blocking property can retard food photooxidation and discoloration^62^. As shown in Table 2, the PC film had UV and visible light transmissions of 44.93 and 81.42%, respectively, suggesting its potential to act as a moderate barrier to UV^63^. CNC/PC nanocomposite films showed lower transmission values across the UV–vis spectrum, where T_280_ and T_600_ decreased with increasing the content of CNC^64,65^. Incorporation of ZnO NPs reduced T_600_ and T_280_ from 70.11 to 29.93% and 28.27 to 4.93%, respectively, due to UV absorption by the uniformly dispersed ZnO NPs^66^ In line with several studies^22,67^, films containing 3% (w/w) ZnO NPs were more opaque (8.52 1/mm) than those containing the maximum CNC concentrations (2.82 1/mm), in consequence to light scattering by ZnO NPs, their size distribution, and refractive indices variation between film ingredients^68^.

**Table 2:** Optical, mechanical, barrier and physicochemical characteristics of the films. Values are presented as means ± SD (*n* = 3) from at least two independent experiments. Different superscript letters within the same column indicate significant differences (*p* < 0.05).

Films	T_600_ (%)	T_280_ (%)	Opacity (1/mm)	Thickness (mm)	TS (MPa)	E (%)	YM (MPa)	Toughness (kJ/m^3^)	WVPx10^−11^ (g.m^−1^.s^−1^.Pa)	MC (%)	WA (%)
PC	81.24 ± 0.80 ^a^	44.93 ± 0.71^a^	1.89 ± 0.09^f^	0.047 ± 0.002^d^	7.53 ± 1.51^d^	7.06 ± 2.23^c^	198.54 ± 4.71^b^	348.79 ± 2.96^g^	4.46 ± 0.23^a^	16.41 ± 2.2^a^	27.33 ± 2.81^bc^
CNC/PC I	77.63 ± 0.55^a^	38.73 ± 0.61^b^	2.27 ± 0.06^ef^	0.048 ± 0.001^d^	7.75 ± 0.96^d^	7.88 ± 2.21^bc^	169.27 ± 7.66^c^	380.35 ± 7.81^g^	2.49 ± 0.24^bc^	13.47 ± 0.99^ab^	28.41 ± 2.91^ab^
CNC/PC II	72.56 ± 0.58^b^	34.71 ± 1.28^c^	2.72 ± 0.06^e^	0.051 ± 0.01 cd	9.12 ± 1.2^ cd^	10.26 ± 1.68^abc^	141.36 ± 1.21^c^	479.19 ± 13.05^f^	2.24 ± 0.01 cd	11.02 ± 0.56^bc^	28.62 ± 0.54^ab^
CNC/PC III	70.11 ± 0.23^b^	28.27 ± 0.60^d^	2.84 ± 0.02^e^	0.053 ± 0.01^bc^	10.48 ± 1.08^bc^	13.21 ± 0.78^a^	141.03 ± 9.95^c^	896.41 ± 14.50^a^	1.86 ± 0.05^d^	10.37 ± 0.44 cd	28.46 ± 1.35^ab^
CNC/PC IIII	63.13 ± 0.80^c^	24.47 ± 0.82^e^	3.64 ± 0.10^d^	0.056 ± 0.003^bc^	8.02 ± 1.05^ cd^	11.1 ± 2.39^ab^	205.27 ± 6.91^b^	558.64 ± 4.66^e^	2.90 ± 0.19^b^	9.76 ± 0.40^cde^	33.03 ± 2.62^a^
ZnO/CNC/PC I	55.39 ± 3.15^d^	14.47 ± 0.45^f^	4.65 ± 0.45^c^	0.054 ± 0.003^bc^	13.01 ± 2.47^ab^	9.22 ± 2.52^abc^	268.61 ± 12.11^a^	820.22 ± 12.27^c^	1.84 ± 0.01^d^	8.84 ± 0.25^cdef^	28.89 ± 1.007^ab^
ZnO/CNC/PC II	41.46 ± 1.94^e^	11.53 ± 1.01^g^	6.90 ± 0.36^b^	0.056 ± 0.003^bc^	13.12 ± 0.86^ab^	9.25 ± 1.42^abc^	275.28 ± 8.68^a^	841.0 ± 18.44^bc^	1.76 ± 0.01^d^	8.23 ± 0.31^def^	27.56 ± 1.04^ab^
ZnO/CNC/PC III	33.7 ± 1.41^f^	7.26 ± 0.94^ h^	8.20 ± 0.31^ab^	0.058 ± 0.001^ab^	13.65 ± 0.56^a^	9.23 ± 2.66^abc^	279.55 ± 2.33^a^	869.22 ± 12.26^ab^	1.11 ± 0.01^e^	7.85 ± 0.35^ef^	23.54 ± 2.23^bc^
ZnO/CNC/PC IIII	29.93 ± 1.05^f^	4.93 ± 0.20^i^	8.52 ± 0.25^a^	0.061 ± 0.005^a^	9.94 ± 0.96^bcd^	8.66 ± 1.15^abc^	264.87 ± 8.10^a^	688.77 ± 11.60^d^	2.04 ± 0.18^ cd^	6.90 ± 0.22^f^	22.01 ± 0.93^c^

The different superscript letters (a–g) within the same column indicate statistically different groups.

#### Thickness and mechanical strength

As indicated in Table 2, the thickness of the studied films varied from 47 to 61 μm owing to the increased solids content. The TS, E%, and YM are the most important and widely measured properties of materials used for packaging applications. The addition of CNC imparted a notable rise in TS values reaching its maximum at CNC concentration of 5% (w/w) (10.48 MPa) compared to PC films (7.53 MPa), in view of the high tensile storage modulus for CNC and its homogenous distribution within the pectin matrix and its high interfacial interactions with polymeric chains pectin matrix^69^. The opposite pattern was demonstrated at higher CNC concentrations (7% w/w), resulting in films with reduced TS of 8.02 MPa. This could be related to CNC aggregation and self-networking, leading to a non-homogeneous distribution into the polymer matrix in agreement with Abdollahi et al.^70^ and Khan et al.^61^, who reported a reduction in TS of alginate- and chitosan-based films at CNC concentrations ˃ 5% (w/w). E% data displayed an increasing trend with CNC concentrations up to 5% (w/w)^27,71^ while YM values was reduced to 141.03 MPa (30% decrease compared to PC film; 198.54 MPa) proving the increased elasticity of the film as deducted from the stress–strain curves (Fig. S2). This is may be due to the interfacial interaction between pectin and CNC that leads to good transfer for the applied stress through CNC/pectin layers. As a result of the increased TS and E% in CNC/ PC films, toughness of the films also increased. The PC film possessed a stored energy of 348.79 kJ/m^3^, this value significantly improved (*P* ˂ 0.05) after CNC incorporation (Table 2) reaching its maximum value of 896.41 kJ/m3 in PC/CNC III film. The poor distribution of CNC at 7% (w/w) was verified by the decrease of elongation by 16% compared to that of films with 5% CNC (w/w), and increase in YM to 205.27 MPa because of the increased potential interactions within each phase at the expense of interfacial interactions between pectin molecules and CNC.

In the ternary systems, TS, YM and toughness were increased notably with increasing the proportions of ZnO NPs to reach the highest value (13.65 MPa, 275.55 MPa and 869.22 kJ/m^3^ for TS, YM and toughness, respectively) at 2% (w/w), attributed to interfacial interactions between ZnO NPs, CNC, and pectin molecules, including the linking between Zn^2+^ ions and the negatively charged groups of pectin chains (COO–) as described in “egg-box”^22^. Agglomeration and recrystallization of the nanostructured metal oxide and subsequent lowering of film matrix cohesiveness may account for the reduction in TS, E%, YM and toughness values at 3% (w/w) ZnO NPs^72^. It is worth mentioning that the TS of ZnO/CNC/PC III film was comparable to that of LDPE and cross-linked PE, despite exhibiting lower E%^73^.

#### Water barrier properties

Water content is a critical parameter that directly influences the rate of microbial spoilage, sensorial properties, and deterioration rates of foods^74^. In the present study, the WVP value for PC film was the highest (4.46 × 10^−11^ g/Pa. s. m) among all tested films. WVP was significantly (*p* < 0.05) lowered by the incorporation of CNC (Table 2) with the least value (1.86 × 10^−11^ g/Pa. s. m) displayed by CNC/PC III film, owing to the reduction of hydrogen bonding with water molecules, and formation of the tortuous pathway^75^. Films containing CNC and different ZnO NPs concentrations showed insignificantly different (*p* > 0.05) WVP, except for ZnO/CNC/PC III film (1.11 × 10^−11^ g/Pa. s. m), which was attributed to the increased film matrix's cohesion caused by the hydrogen bonding and interactions between nanostructured fillers and pectin chains^72^ as supported by the SEM data (Fig. 4). Films containing higher ZnO NPs (3% w/w) were more permeable to water vapor (2.04 × 10^−11^ g/Pa. s. m), which could be reasoned to agglomeration and forming voids and pores disrupting the polymeric matrix^76^. Nevertheless, the WVP data provides evidence for the applicability and performance of the active films that may compete with frequently used commercial materials such as cellophane (WVP of 8.4 × 10^−11^ g/Pa. s. m)^77^.

#### MC and WA

MC and WA of films are significant determinants that affect the rheological and mechanical properties, especially for hydrophilic polymers. Moreover, the water sensitivity of pectin-based films limits their application to certain food commodities^78^. Results in Table 2 showed that pectin nanocomposite films had less moisture than that of PC films (16.41%), probably related to the reduced space available for water mobility after fillers interaction with pectin matrix, and thus lowering the moisture content of the composite film^56,79^. WA of active films were directly proportional to CNC content, however, WA values for films containing 1, 3, or 5% (w/w) of CNC concentrations were insignificantly different (*p* > 0.05). The maximum WA (32.0%) recorded for films containing 7% (w/w) CNC was ascribed to CNC aggregation at the expense of interfacial interactions with pectin; consequently, pectin groups were available to interact with water molecules^27^. Contradictory results by Ahmadi et al.^60^ reported that the incorporation of cellulose nanofibers at a concentration of 7.5% (w/w) in gelatin films resulted in a noticeable decrease in water adsorption. On the other hand, incorporating ZnO NPs into CNC/pectin nanocomposite films caused a decline in WA values, in agreement with studies on bacterial cellulose and pectin-based films^44,67^.

#### Thermal stability

**Figure 5:**
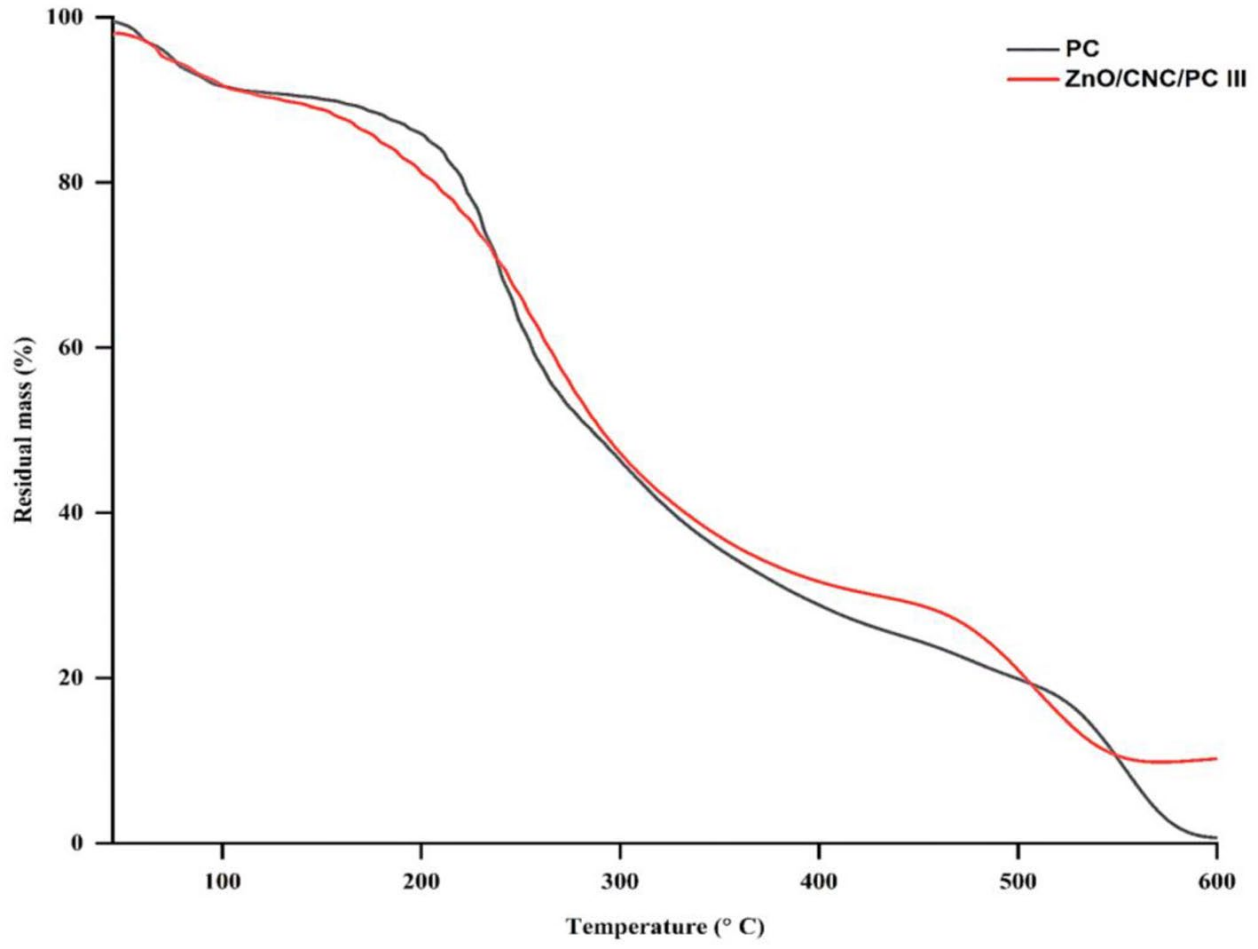
TGA thermograms of PC and ZnO/CNC/PC III films.

Figure 5 depicts the 3-stage TGA thermograms of PC and ZnO/CNC/P**C** III films. The first stage (45–130 °C) corresponds to the loss of water bound to hydrophilic groups in control and active film (8.88 and 7.36%), respectively^62^ in support of the MC data (Table 2). In the second stage (150–310 °C), PC films showed a weight loss of 48.29%, versus 44.78% in ZnO/CNC/P**C** III films at 160–320 °C, due to depolymerization of pectin chains and PEG_400_ degradation^80^. The temperature of maximum weight loss (T*max*) at the same stage in ZnO/CNC/PC III films exceeded that of PC films by 20 °C. A plausible explanation is that ZnO NPs may act as heat insulators during thermal transport^11^, coupled with the strong interfacial interactions between nanofillers and pectin chains, causing depolymerization difficulty^57^. The third stage in both films (T > 450 °C) was associated with the thermal breakdown of charred residues to low molecular weight gaseous products^81^. The ash content for PC and ZnO/CNC/P**C** III films at 600 °C was 1.81 and 10.02%, respectively, due to inorganic filler represented by ZnO NPs^63^.

### Migration of Zn^+2^

As far as we know, published data describing the migration of ZnO NPs from pectin-based films to food or food simulants is limited. According to EFSA, ZnO NPs don’t migrate in the nanoform, so evaluation was based on the detection of soluble ionic zinc^23^. We propose the first evaluation of Zn^+2^ migration into roumy cheese samples wrapped with the active ZnO/CNC/PC III film. Zn^+2^ initially detected in cheese samples was 60 ± 1.15 mg/kg, which was higher than that obtained by Deeb^82^ and Mohammed^83^. In the present work, 3 mg/kg of Zn^+2^ has been confirmed to migrate from the nanocomposite film (containing 7.5 mg zinc) after 5 days of refrigerated storage. This concentration meets the specific migration limits (5 mg/kg food) approved by the European Plastics Regulation (EU) 2016/1416, amending and correcting Regulation (EU) 10/2011 for food-contact items^84^. Comparable results for percentages of migrated Zn^+2^ were reported by Souza et al.^36^ in chicken fillet samples packaged with chitosan films containing 1–2% ZnO NPs after 11 days of storage. A higher migration rate (15.5 mg/kg) from PLA-ZnO film composites was determined in fish fillets after 16 days of storage^85^. It should be pointed out that the amount of migratable zinc is governed by many factors such as the characteristics of the NPs (size, shape), polymer nature, and food conditions (pH, composition)^86^.

### Wrapping experiment

**Figure 6:**
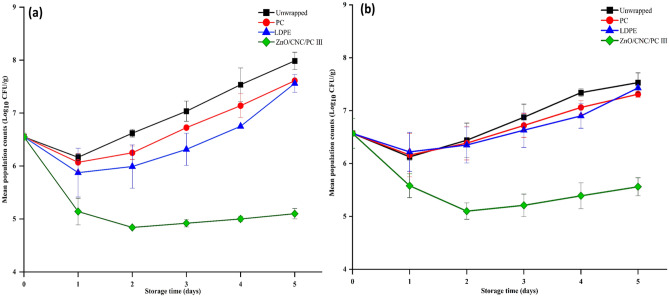
Growth behavior of *S. aureus* (**a**) and *S. enterica* (**b**) on unwrapped, LDPE-, PC-, and ZnO/CNC/PC III wrapped roumy cheese.

ZnO/PC/CNC III film was selected in the wrapping experiment based on the promising antimicrobial activity of the corresponding FFS, and characterization data of mechanical and water barrier experiments. Growth inhibition of *S. aureus* and *S. enterica* on contaminated cheese slices wrapped with the active and control films stored at 7 °C for 5 days are illustrated in Fig. 6. Results were compared to those of samples wrapped with commercial LDPE films. After 1 day of storage, population counts of *S*. *aureus* in cheese slices wrapped with the active film were reduced by 1.41 log CFU/g versus 0.39, 0.48, and 0.65 log CFU/g for unwrapped, PC-wrapped, and LDPE-wrapped cheese samples, respectively. On day 5, the active film was able to inhibit the growth of S. aureus populations by 1.45 log unit, whereas the final population counts reached ~ 8 log CFU/g (1.4 log units increase) in unwrapped cheese samples (Fig. 6a), probably associated with the slow release of Zn^2+^ , cell wall penetration, and reaction with the cytoplasmic content causing bacterial death. Furthermore, ZnO NPs can possibly generate reactive oxygen species that may damage the bacterial cell membrane^87^. A study by Amjadi et al.^42^ reported a 2-log reduction in *S. aureus* counts after 12 days of refrigerated storage of chicken fillet samples coated with gelatin-chitin nanofibril-ZnO NPs films. Vacuum packaging of a ready-to-eat turkey using pullulan-xanthan gum-ZnO NPs composite-based film was found to support *S. aureus* counts that were 3 log units less than those detected in control samples after 7 days of storage^37^.

In general, growth reduction of *S. enterica* in active film-wrapped samples was lower than that of *S. aureus,* mainly based on differences in susceptibility towards ZnO NPs. *S. enterica* population counts exhibited a similar reduction pattern in all samples (Fig. 6b). Population counts were reduced after 24 h, followed by a gradual increase until the end of the storage period except for the sample wrapped with the active film. By day 5, final counts recorded in unwrapped cheese slice samples, samples wrapped with PC, LDPE or the active films were 7.53, 7.31, 7.43 and 5.56 log CFU/g, respectively. This result was in partial agreement with data found by Souza et al.^36^, where the growth of Gram-negative enterobacteriaceae was inhibited (⁓ 2.4 log reduction) in raw poultry meat samples coated with chitosan films containing 2% ZnO NPs and chill-stored for 7 days.

## Conclusion

In the present study, ZnO NPs were prepared with high purity using the co-precipitation method and were incorporated for the first time with CNC in pectin-based matrix to fabricate active films by the solvent-casting technique. The optimal active films (3% w/v pectin) contained 2 and 5% (w/w) ZnO NPs and CNC, respectively. The nanofillers were uniformly distributed in the pectin matrix as indicated by the SEM micrographs, and their interactions were confirmed by FTIR spectra. The active ternary films showed enhanced UV-blocking, TS, thermal, and water vapor barrier properties exceeding PC and CNC-containing films. The optimal active films demonstrated their efficiency as wrapping materials for artificially contaminated cheese samples, where the growth of *S. enterica* and *S. aureus* populations were significantly reduced compared to those in PC- and LDPE-wrapped samples after 5 days of storage at 7 °C. The high IC_50_ value of the active FFS (24.61% of FFS containing 145 µg ZnO/mL) against normal cell lines as analyzed by MTT assay, and the low specific migration of Zn^2+^ to food samples, inferred the safety of the prepared active films. Overall, the results may be of technological significance that warrant potential application of the novel active films as safe and eco-friendly packaging material.

## Data avialability

Correspondence and requests for materials should be addressed to **M.R.S**. based o reseonable request.

## Supplementary Information

Supplementary Information.
