# Supplementary material for: Novel pectin-based nanocomposite film for active food packaging applications
Source: Sci Rep. 2022 Nov 30;12:20673. doi: 10.1038/s41598-022-25192-4 (PMC9712656; doi:10.1038/s41598-022-25192-4)
Supplement: Supplementary file 1 — Supplementary Information. [file 41598_2022_25192_MOESM1_ESM.pdf]

Supplementary Material

Novel pectin-based nanocomposite film for active food packaging applications

Muhammed R. Sharaby[1,2,*] , Emad A. Soliman[3], Adel B. Abdel-Rahman[4],

Ahmed Osman[1,5], and Rowaida Khalil[2]

[1]Basic and Applied Sciences (BAS) Institute, Egypt-Japan University of Science and Technology (E-JUST), New Borg El-Arab city, Alexandria 21934, Egypt.

[2]Botany and Microbiology Department, Faculty of Science, Alexandria University, Alexandria 21511, Egypt.

[3]Polymeric Materials Research Department, Advanced Technology and New Materials Research Institute (ATNMRI), City of Scientific Research and Technological Applications (SRTA-City), New Borg El-Arab City, Alexandria 21934, Egypt.

[5]Department of Electronics and Communications Engineering, Egypt-Japan University of Science and Technology, New Borg El-Arab city, Alexandria 21934, Egypt

[5]Department of Biochemistry, Faculty of Science, Ain Shams University, Cairo, Egypt.

[*]Corresponding author: Muhammed R. Sharaby (muhammed.sharaby@ejust.edu.eg; muhammedsharaby@alexu.edu.eg)

This file includes:

Table S1

Figure S1

Figure S2

FFSs	Diameters of inhibition zones (mm)					
	Staphylococcus aureus	***E. coli* O157:H7**	***Salmonella enterica***	***Klebsiella pneumoniae***	***Pseudomonas aeruginosa***	***Proteus mirabilis***
**PC**	0^{c}	0^{d}	0^{d}	0^{c}	0^{d}	0^{b}
**CNC/PC I**	0^{c}	0^{d}	0^{d}	0^{c}	0^{d}	0^{b}
**CNC/PC II**	0^{c}	0^{d}	0^{d}	0^{c}	0^{d}	0^{b}
**CNC/PC III**	0^{c}	0^{d}	0^{d}	0^{c}	0^{d}	0^{b}
**CNC/ PC IIII**	0^{c}	0^{d}	0^{d}	0^{c}	0^{d}	0^{b}
**ZnO/CNC/PC I**	12.50 ± 0.70^{b}	8.0 ± 0^{c}	8.0 ± 0.70^{c}	0^{c}	7.50 ± 1.0^{c}	0^{b}
**ZnO/CNC/PC II**	15.0 ± 0.70^{ab}	12.0 ± 0.57^{b}	13.50 ± 0.57^{b}	0^{c}	12.0 ± 1.0^{b}	0^{b}
**ZnO/CNC/PC III**	17.0 ± 1.41^{ab}	15.0 ± 0.70^{a}	15.50 ± 0.70^{ab}	8.50 ± 1.41^{b}	15.50 ± 2.12^{ab}	0^{b}
**ZnO/CNC/PC IIII**	19.0 ± 2.21^{a}	16.0 ± 0^{a}	18.0 ± 1.41^{a}	11.50 ± 0.70^{a}	17.50 ± 0.70^{a}	8.50 ± 1.41^{a}

Table S1 . AMA of pectin-based FFSs against different bacterial strains. Values are presented as means ± SD ($n = 3$) from at least two independent experiments. Different superscript letters within the same column indicate significant differences ($p < 0.05$).

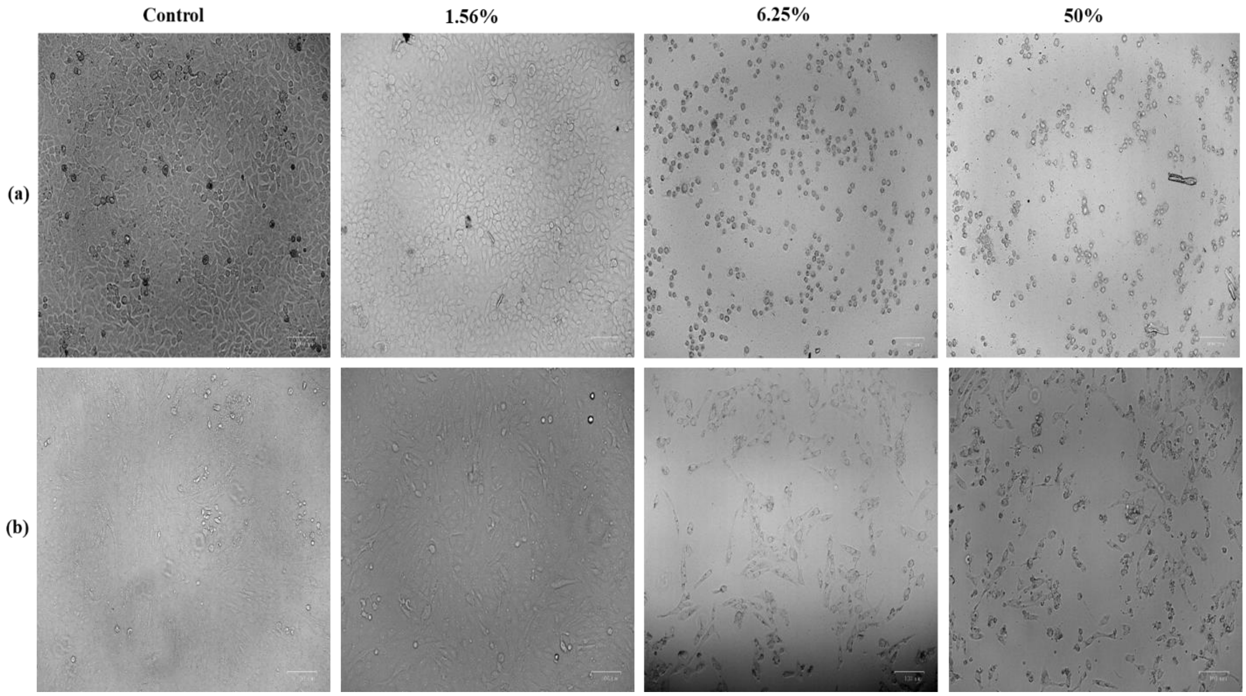

Figure S1: Morphology of Caco-2 (a) and WI-38 (b) treated with different concentrations of FFSs.

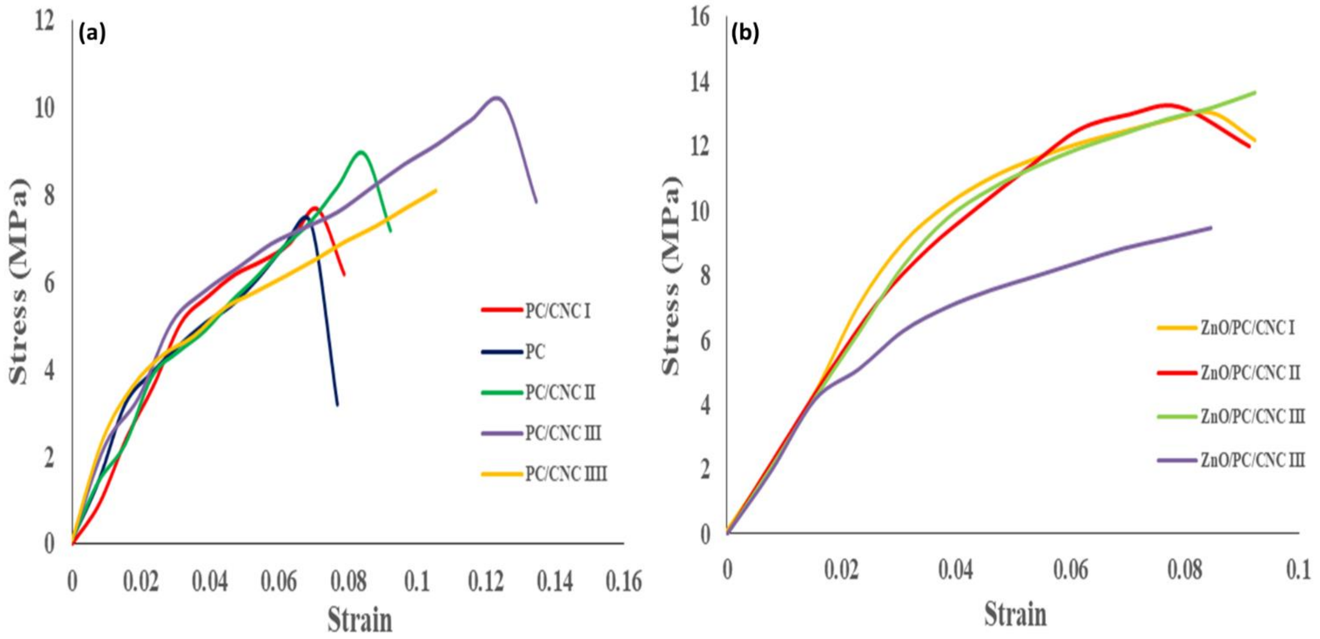

Figure S2: Stress-strain curves of one replicate of PC and PC/CNC (a) and ZnO/PC/CNC (b) nanocomposites.
